# Cellular and Molecular Mechanisms Activated by a Left Ventricular Assist Device

**DOI:** 10.3390/ijms25010288

**Published:** 2023-12-24

**Authors:** Antonella Galeone, Cinzia Buccoliero, Barbara Barile, Grazia Paola Nicchia, Francesco Onorati, Giovanni Battista Luciani, Giacomina Brunetti

**Affiliations:** 1 Department of Surgery, Dentistry, Pediatrics and Gynecology, Division of Cardiac Surgery, University of Verona, 37129 Verona, Italy; antonella.galeone@univr.it (A.G.); francesco.onorati@univr.it (F.O.); giovanni.luciani@univr.it (G.B.L.); 2 Department of Biosciences, Biotechnologies and Environment, University of Bari Aldo Moro, 70125 Bari, Italy; cinzia.buccoliero@uniba.it (C.B.); barbara.barile@uniba.it (B.B.); graziapaola.nicchia@uniba.it (G.P.N.)

**Keywords:** LVAD, inflammation, von Willebrand disease, malnutrition

## Abstract

Left ventricular assist devices (LVADs) represent the final treatment for patients with end-stage heart failure (HF) not eligible for transplantation. Although LVAD design has been further improved in the last decade, their use is associated with different complications. Specifically, inflammation, fibrosis, bleeding events, right ventricular failure, and aortic valve regurgitation may occur. In addition, reverse remodeling is associated with substantial cellular and molecular changes of the failing myocardium during LVAD support with positive effects on patients’ health. All these processes also lead to the identification of biomarkers identifying LVAD patients as having an augmented risk of developing associated adverse events, thus highlighting the possibility of identifying new therapeutic targets. Additionally, it has been reported that LVAD complications could cause or exacerbate a state of malnutrition, suggesting that, with an adjustment in nutrition, the general health of these patients could be improved.

## 1. Introduction

Heart failure (HF) represents one of the most widespread public health problems, involving about 100 million people worldwide, with 1-year mortality evaluated at 15–30% [1]. End-stage HF is characterized by poor heart function, with consequent development of discomfort and dyspnea even in the absence of physical activity [2]. Unfortunately, HF medications grant only transient relief. Heart transplantation (HT) represents the gold-standard treatment for patients with advanced HF [3], as it is associated with improved survival and quality of life in these patients [4]. However, its wider application is limited by the scarce availability of suitable organ donors. The huge discrepancy existing between the limited availability of donor hearts and the growing number of patients with HF whose condition deteriorates while on the HT waiting list or suffering end-organ dysfunction at listing, has also led to an increase in the use of marginal donors [5] and left ventricular assist devices (LVADs) as a bridge to transplant therapy (BTT) [6]. LVADs pump blood from the left ventricle (LV) to the aortic root, thus representing a mechanical pump that works in parallel with the native LV. Implantable LVADs have also emerged as a new treatment option for advanced HF as a destination therapy (DT) for patients either too old or ineligible for HT, meaning that the patient is expected to live with the device until death. Previous studies demonstrated fewer symptoms and better event-free survival and quality of life with LVAD-DT compared to optimal medical management [7]. In the current era, DT is the predominant indication for LVAD implantation, with 78% of LVADs implanted as a DT in 2020, a dramatic increase from 49.5% in 2017 [8]. The marked increase in DT implants since 2017 is coincident with the approval of the fully magnetically levitated centrifugal-flow device HeartMate 3 (HM3) in 2018; third-generation implantable continuous-flow (CF) LVADs, incorporating improved pump technologies, have better pump performance and lower adverse event profiles [9].

**Figure 1:**
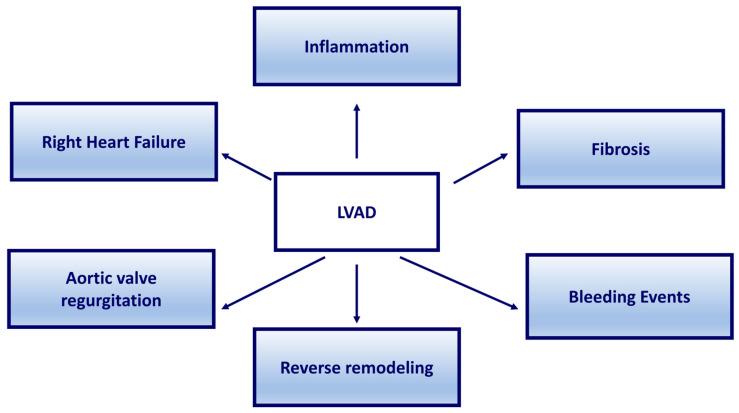
Mechanisms associated with LVAD implantation.

In this review, we consistently aim to detail the major adverse events associated with LVAD implantation, including bleeding, thrombosis, ischemic and hemorrhagic strokes, renal impairment, multi-organ failure, and infections, which have been the primary causes of death in some series [6,10,11]. These causes of morbidity and mortality have been linked to the activation of inflammatory cascades [12]. Furthermore, we describe how LVAD complications can cause or exacerbate a state of malnutrition (Figure 1).

## 2. Inflammation

HF is characterized by the activation of innate and adaptive immune systems [13] with the release of inflammatory cytokines, notably tumor necrosis factor alpha (TNF-α) [14,15] and interleukin-6 (IL-6) [16].

Many studies focused on changes in levels of inflammatory markers during long-term LVAD support. Mechanical unloading with LVAD is associated with significant reductions in myocardial proinflammatory cytokine levels, including TNF-α, IL (interleukin)-6, IL-1β, Fas, and FLICE (FADD-Like ICE) [17,18,19].

Although a significant reduction in brain natriuretic peptide (BNP) levels, an indicator of reduced myocardial stress, has been observed, circulating levels of inflammatory markers such as TNF-α, IL-8, monocyte chemoattractant protein-1 (MCP-1), interferon γ-induced protein, and C-reactive protein (CRP) were significantly elevated after LVAD support [20,21,22,23,24]. This persistent inflammatory state may reflect a systemic foreign body response and a response to ongoing damage to blood elements caused by the pump.

Increased TNF-α levels in patients with LVAD contribute to pericyte apoptosis and angiopoietin-1 suppression resulting in vascular destabilization [25]. Moreover, elevated TNF-α levels have been associated with a major susceptibility to nonsurgical bleeding [25] and may play a central role in LVAD-related angiodysplasia [26]. IL-6 is a cytokine with pleiotropic activity involved in inflammation, immunity, and disease [27]. Interleukin-6 acts on acquired immunity, both B and T lymphocytes. An increase in IL-6 levels induces stimulation of B cells, resulting in the augmented antibody production of CD4 and CD8 T cells, causing over-differentiation of Th17 and cytotoxic T cells, respectively, and a reduction in T reg cell differentiation [27]. Moreover, after LVAD implantation, T-cell proliferative activity increased with defective responses after activation [28]; this results in an increased occurrence of candida infections in 28% of patients compared to controls [28]. The surface expression of CD95, a molecule associated with the cell apoptosis pathway, in patients receiving LVAD was significantly increased in both CD4 (*p* < 0.001) and CD8 (*p* < 0.001) T cells [28] than controls.

After LVAD implantation, the increase in inflammation activation may result in an augmented risk of multiple organ failure syndrome (MOFS). Elevated amounts of IL-6, after LVAD implantation, correlate with longer hospital stays and sequential organ failure assessment (SOFA) after 1 week, which in turn is associated with greater multiorgan dysfunction [29]. Moreover, Caruso and colleagues demonstrated the relationship between systemic inflammatory response [IL-6, IL-8 and C-reactive protein (CRP)] after LVAD implantation and high risk for MOFS in a cohort of 23 patients [30]. Furthermore, Sundararajan et al. highlighted the correlation between neutrophil–lymphocyte ratio (NLR) and mortality during LVAD implantation [31]. NLR can be altered during an inflammatory process, in favor of an increase; so, it can be used as hematological marker for a prognostic utility use [32]. Consistently, Solanki et al. reported that numerous patients undergoing LVAD implantation showed enhanced NLR at the time of surgery. This ratio normalized over time in some patients, and about half of the patients displayed normal NLR 90 days after LVAD implantation. At 90 days after LVAD implantation, an increased NLR was linked to worse overall survival [33]. Otherwise, Diakos et al. found that survivors showed reduced NLR levels prior to LVAD implantation. Both survivors and non-survivors displayed increased IL6 levels, which decreased after implantation only in survivors, whereas it further increased in non-survivors [34].

In a failing human heart, immunohistochemical staining for CD68 before and after LVAD implantation did not show a statistically significant variation of resident cardiac macrophage [35,36]. Otherwise, the switch of macrophage phenotype is evident with mechanical unloading. Isolated cardiac macrophages displayed decreased levels of pro-fibrotic M2 macrophage genes, including TGM2, KLF-4, and MRC1, with a trend toward the augmentation of pro-inflammatory M1 macrophage genes such as TNF-α and IL-1β following mechanical unloading [35]. In macrophages, LVAD implantation induced a particular profile with a statistically significant decrease in mRNA levels of matrix metalloproteinase (MMP) 2, which is involved in the persistent myocardial fibrosis associated with mechanical unloading. 

Margulies et al. demonstrated that only 238 transcripts dysregulated in HF showed a statistically significant change also after LVAD implantation [37]. Only a small proportion of HF-dysregulated genes reached the control levels following LVAD implantation; however, different mRNAs of pro-inflammatory pathways are dysregulated and included (C/EBPβ [enhancer-binding protein beta], NFKBIA [NFKB inhibitor alpha], CXCL12 [C-X-C motif chemokine ligand 12], CCL2 [C-C motif chemokine ligand 2], and CD14). In the same conditions, the oxidative stress pathways (MT1F [metallothionein 1F] and MT1X [metallothionein 1X]) were also significantly modified [38]. Macrophage lineage activation was demonstrated by an increase in cytokine and coagulation factor release [39]. Notably, LVAD implantation resulted in thromboembolic events in 30% of patients [40]. Walenga and colleagues demonstrated for the first time that a thrombosis event associated with LVAD implantation causes upregulation of the inflammatory process compared with classical LVADs without any thrombosis event [41]. It is noteworthy that thrombosis events associated with LVAD implantation caused a 3- to 4-fold increase in CRP compared with other LVAD patients [41].

## 3. Fibrosis

HF is associated with fibroblast activation and deposition of extra-cellular matrix (ECM) proteins. Previous reports showed no change or significant increase in myocardial fibrosis after LVAD support [42]. LVAD induces an increase in the total myocardial collagen content and in the cross-linked collagen causing increased myocardial stiffness [43]. Additionally, myocardial mRNA levels of MMP-2 and MMP-9 are downregulated during LVAD support with reduced circulating levels of MMP-9 [44]. MMP-1 levels are also reduced, while TIMP-1 protein levels are increased, with a decrease in MMP1/TIMP-1 ratio, which favors collagen deposition [45]. Different fibrosis biomarkers have been identified, such as ST2, galectin-3, and growth differentiation factor-15.

### 3.1. ST2

ST2 is a member of the interleukin-1 receptor family that can be found as soluble and membrane-bound forms [46]. Its ligand is represented by IL-33, and the IL-33/ST2 interaction has a protective effect against fibrosis and myocardial hypertrophy [47]. Soluble ST2 (sST2) binds and inactivates IL-33 with consequent blocking of their interaction, thus determining the loss of the IL-33 cardioprotective function in HF. sST2 is produced by endothelial cells as well as human myocardium and represents an independent risk factors for mortality in HF, and its expression is reduced by treatment [48,49].

In an LVAD study involving 38 patients, serial ST2 serum evaluations were performed at pre-implantation and at 1, 3, and 6 months after LVAD implantation. ST2 levels were significantly increased in end-stage HF before LVAD implantation and reduced during LVAD support to basal physiological levels in 3 months in the majority of patients, thus suggesting that ST2 may represent a useful biomarker to monitor therapy in end-stage HF [50]. Although ST2 levels have been recognized as useful markers for HF prognosis and monitoring by the FDA and American College of Cardiology/American Heart Association [51,52], the role of ST2 levels in assessing myocardial recovery in LVAD patients required evaluation in large clinical trials [52].

### 3.2. Galectin-3

Galectins, members of the lectin family, are characterized by their binding to ligands presenting β-galactoside structures [53]. This interaction involves the galectin carbohydrate recognition domains (CRDs). Galectin-3 is coded by the LGALS3 gene on chromosome 14 and can be found in different compartments in the body. Galectin-3 is a defined “chimera” and can be organized in different forms: (1) the primary form contains the N-terminal domain with 120 amino acids leading to oligomer formation as well as the exposure of the molecule on the cell membrane [54]; a polypeptide chain of 250 amino acids; the collagen alpha-like medial sequence that can be cleaved by metalloproteinases [54]; the C-terminal domain with 130 amino acids [54]; (2) in the secondary form, CRD is arranged as an antiparallel β-pleated sheet, important for conferring it flexibility and resilience to stretching [54]; (3) a globular tertiary form; (4) a quaternary form that can be either mono- or multimeric depending on the concentration: if the concentrations are reduced, monomers can be observed, supporting intercellular adhesion through the interaction with integrins on other cells; differently, if the concentrations are elevated, multimers act to mediate intercellular adhesion.

The complexity of its mechanism of action is due to its interaction with numerous proteins of the cell membrane, extracellular matrix, biological fluids, and intracellularity. Hence, it has been described as being implicating in numerous biological and pathological events [54,55,56,57], such as inflammatory and fibrotic diseases. Galectin-3 influences the beginning and expansion of the acute inflammatory response by attracting macrophages into injury sites and thus exacerbates chronic inflammation by activating proinflammatory pathways, leading to fibrosis, a development that perpetuates inflammation and abnormal tissue repair.

In heart disease, Galectin-3 is produced by activated cardiac macrophages with pro-fibrotic properties and thus involved in adverse remodeling and hypertrophy. High levels of Galectin-3 have been detected in HF patients with adverse outcomes [58]. Interestingly, Galectin-3 elevated levels were demonstrated at the time of LVAD implantation. Moreover, patients with LVAD and multiple-organ failure displayed higher Galectin-3 levels at the time of LVAD implantation compared to patients that had a successful transplantation performed [59,60]. Higher levels of Galectin-3 have been linked to increased mortality after LVAD implantation [59]. These findings were supported by another study involving 151 patients who received a LVAD [61].

### 3.3. Growth Differentiation Factor-15 (GDF-15)

GDF-15, member of the transforming growth factor beta superfamily, is emerging as a biomarker of fibrosis, HF, and cardiac remodeling [62,63]. Circulating GDF-15 and myocardial levels were evaluated before and at 1, 3, and 6 months post-LVAD implantation, at the time of heart transplantation, or following LVAD explantation. Interestingly, already one month post-LVAD implantation, GDF-15 levels were reduced compared to pre-implantation levels and remained stable. In addition, 96% of the end-stage HF patients showed high GDF-15 levels before LVAD implantation, and, interestingly, its levels remained high only in 25% of patients at 6 months post-LVAD implantation. In LVAD patients receiving antithrombotic therapy, the GDF-15 levels are inversely related to residual platelet reactivity [64]. These results can be explained by the recent discoveries demonstrating that GDF-15 partially inhibits platelet integrin activation and thrombus formation [65,66]. GDF-15 has been consistently linked to bleeding events in different kinds of morbidity; thus, it has been recommended as a new biomarker for bleeding complications [67,68] that are also a typical complication of LVAD.

## 4. Bleeding Events

Despite the miniaturization of the device, the evolution of pump technology, and the improvements in the latest generation pump’s performance, LVADs establish some negative effects on the endothelium, such as augmented shear stress with consequent destruction of the equilibrium regulating the balance of angiogenic factors with the resulting deregulated angiogenesis [69]. Although the mechanisms should be clarified, it has been reported that the altered shear stress determines an increased degradation of the von Willebrand factor (vWF). 

### 4.1. von Willebrand Factor (vWF) 

It is a large multimeric glycoprotein produced by endothelial cells and megakaryocytes that is released as a consequence of hemorrhagic events, thus participating in the formation of a platelet plug in the vessel. vWF presents binding sites for the receptor glycoprotein Ib (GP1b) on non-activated platelets and the glycoprotein IIb/IIIa (GPIIb/IIIa) receptor on activated platelets. The activated endothelium generates a locally high concentration of vWF that, following the binding with the platelet, starts the formation of thrombus [70]. vWF also works as a carrier for Factor VIII, thus prolonging its half-life and degradation [70,71]. The multimer size is important for determining hemostatic capability, which decreases with a reduction in its size. Following its release by endothelial cells and thanks to cleavage by ADAMTS13 [72], vWF circulates but is progressively reduced in size by the same cleavator. The cleaved functional monomers represent the circulating pool and can be activated by shear conditions or factor VIII and platelets [73,74,75]. 

Additionally, vWF represents a strong regulator of angiogenesis. Consistently, in endothelial cells, it modulates the expression of angiopoietin-2 (Ang-2), which promotes angiogenesis through VEGFR2 signaling [76].

On smooth vascular muscle cells, vWF interaction with α_V_β_3_ determines the cell recruitment, consequently vWF loss alters vessel formation with thin-walled, fragile networks with a tendency to rupture and produce spontaneous bleeding [77].

### 4.2. von Willebrand Disease in LVAD

It has been found that LVADs alter the shear stress by at least one order of magnitude [78], thus exposing vWF to unfolding and activating the cleavage with a consequent decrease in vWF multimers and activity, which therefore leads to spontaneous muco-cutaneous or gastrointestinal bleeding episodes as well as excessive bleeding after surgery or trauma. Over 50% of patients with vWF disease showed gastrointestinal bleeding [79]. Recently, it has been reported that LVAD patients show high levels of Ang-2 in venous endothelial cells, which is associated with increasing in vitro angiogenesis that can be inhibited by an Ang-2 inhibitor antibody [80]. To block vWF disease, different approaches have been used [69].

## 5. Aortic Valve Regurgitation after LVAD Implantation

LVAD implantation has many effects on aortic valve function and histology, including important changes in valvular interstitial cells (VICs) and extracellular matrix. Between 25% and 30% of patients assisted with CF LVADs develop aortic regurgitation (AR) within the first year after implantation [81], which is associated with a poor prognosis [82]. 

The structural changes in the aortic cusps, the altered aortic root biomechanics, and the left ventricular unloading promote cusp remodeling and commissural fusion and contribute to the development and progression of aortic valve disease. Hemodynamics are profoundly altered after LVAD implantation. LVADs decrease the LV loading and the ventricular wall stress, entailing reverse remodeling and a reduction in the size of the ventricular cavity. The aortic valve and aortic root are subjected to a constant high-pressure load, which induces deterioration and remodeling (including collagen proliferation) of the aortic valve, less frequent opening of the aortic valve, and dilatation of the aortic root and contributes to AR progression [83,84].

Rose et al. first described partial fusion of the aortic valve in four of six patients supported with a pulsatile LVAD, all being associated with thrombus, either active or organized [85].

These results were later confirmed by Connelly et al., who described aortic commissural fusion in 17 of 33 patients supported with pulsatile LVADs [86].

Samuels et al. reported on the microscopic examination of the valve across the area of fusion and showed that the fusion tissue was composed of myxomatous granulation tissue adherent to the inflow aspect of the left and noncoronary cusps, while mineralization was absent [87]. The authors attributed valve degeneration following LVAD implantation to systemic pressure-related changes induced by turbulent blood backflow from the outflow cannula onto a closed valve. 

The exact mechanism of aortic valve commissural fusion is not yet completely understood, but it has been hypothesized that it could result from prolonged leaflet coaptation due to poor or no antegrade flow, a finding also seen in patients with chronic HF with decreased cardiac output [86].

Some studies have described morphological and histological changes in the aortic valve tissue of LVAD recipients in terms of valve thickening, collagen accumulation, inflammatory cell infiltration, and activation of VICs by increasing amounts of alpha-smooth muscle actin (α-SMA) with conflicting results.

Hata et al. reported on the aortic valve pathology of 35 hearts removed at the time of transplantation of 31 patients supported with an extracorporeal pulsatile device, 1 patient with an implantable pulsatile device, and 3 patients supported with an implantable non-pulsatile device [88].

Pathological analysis showed that more than 45% of patients had commissural fusion, and there was no predilection for fusion of specific cusps. Leaflet curling and shortening were also observed in more than 60% of the patients. Histological examination revealed that the aortic valve leaflets had become thinner in all patients, ranging from 120 to 1400 µm compared with the normal value of 1500–2000 µm. The aortic wall and the left ventricular wall had also become thinner in most patients. Microscopically, dense collagen accumulation in the spongiosa layer was observed in some patients, while there was no significant sign of inflammation in the leaflets. There was no correlation between aortic valve thickness, aortic annulus dimension, or aortic wall thickness and LVAD support duration, AR grade, or cardiac function. A closed or less frequently opening aortic valve was a significant predictor of late-mild or mild-to-moderate AR. On the contrary, other parameters such as sex, age, LVAD support duration, commissural fusion, cusp thickness, aortic annular dilatation, and aortic wall thickness were not significant predictors of mild or mild-to-moderate AR after long-term LVAD support using logistic regression analysis. Additionally, there was no correlation between commissural fusion and sex, age, LVAD support duration, cusp thickness, aortic valve opening, or types of LVAD [88].

Saito et al. performed a retrospective histological analysis of 38 explanted hearts supported with CF-LVAD from patients who received HT and showed that longer CF-LVAD support duration correlated with a thinner aortic valve’s ventricularis layer [89].

Retrograde flow stress produced by the ascending aortic graft of the CF-LVAD causes the aortic valve to remain in the closed position and the involution of the ventricularis layer of the aortic cusp. The normal aortic valve receives its oxygen supply mostly by diffusion from the surface of the valve [90]. Approximately 30% of the base region and 3% of the rest of the cusp region are vascularized [91].

When the cusp remains closed, the diffusion component is blocked, and only microcirculation can supply the oxygen to the valve. Longer coaptation time makes oxygen supply to the distal aortic cusp more difficult, inducing structural changes due to ischemic processes [89].

Van Rijswijk et al. evaluated 21 aortic valves of patients on long-term CF-LVAD support after HT or at autopsy [92], showing that despite the fact that total leaflet thickness after LVAD support was similar to the control group, the ventricularis layer in the leaflet belly appeared significantly thickened. Overall cell density was enhanced in LVAD patients with respect to the control group, due to a cell density augmentation in the ventricularis layer. Cell density was negatively associated with age. In the spongiosa and ventricularis layers after LVAD support, cell proliferation following Ki-67 labeling was enhanced. In the same study, a significant increase in myofibroblast markers αSMA and calponin was reported in the ventricularis layer after LVAD support, suggesting an increase in VIC activation. αSMA-positive cells were localized mainly in the ventricularis layer and occasionally observed in the fibrosa layer. The presence of αSMA in the leaflets was positively linked to the duration of LVAD support and negatively to age. The authors also showed inflammatory infiltration and activation of repair mechanisms. CD68-positive macrophages were detected in all layers of the aortic leaflets and were significantly increased after LVAD support. The M2-macrophage marker CD163 showed a significant increase in the ventricularis layer of the LVAD patients. M2 macrophages are a cell population with anti-inflammatory properties and are involved in tissue repair [93].

The CD45 leukocyte common antigen was significantly augmented in the spongiosa and ventricularis layers after LVAD support. Few CD3-positive T cells were observed in any valves, while no myeloperoxidase-positive neutrophils were detected in either group [92].

On the contrary, in the study of Martina et al., their histopathological examination showed that 11 of the 19 (58%) aortic valves had a fusion of single or multiple commissures, with varying changes in the valve layer structure, but without evidence of inflammatory infiltration at the site of fusion, suggesting that the fusion process is not driven by inflammation [94].

In a recent study, aortic valves were collected from 16 LVAD and 6 non-LVAD patients at time of HT and analyzed using biaxial mechanical tensile testing, mass spectrometry-based proteomics, and transmission electron microscopy to assess ultrastructure [95].

Aortic valves in LVAD patients showed decreased compliance and increased stiffness compared to non-LVAD patients. Additionally, in the aortic valves of LVAD patients, the expression of proteins related to valve activation and injury, such as proteins associated with actin and myosin, immune signaling, oxidative stress, and transforming growth factor beta (TGF-β), were increased compared to non-LVAD patients [95].

Barth et al. evaluated the impact of LVAD support on structural and molecular alterations of the aortic valve [96].

The authors analyzed aortic valves from 63 patients undergoing HT for end-stage HF with (*n* = 22) and without (*n* = 41) previous LVAD implantation. Calcification and markers of remodeling, chondro-osteogenic differentiation, and inflammation were evaluated using computed tomography, mRNA analysis, histology, and immunohistochemistry. Expression of matrix metalloproteinase (MMP)-9, α-SMA, actin, and osteopontin (OPN) were significantly upregulated in the aortic valves of LVAD patients. Histological appearance of the aortic valve was similar in patients with or without LVAD, and computed tomography-based analysis showed no significant difference in tissue calcification. Expression of interferon gamma (IFN-γ), IL-1 beta, and TNF-α was significantly upregulated in the aortic valves of LVAD patients without concomitant inflammatory cell infiltration. Expression of MMP-2 and TGF-β was negatively correlated with the duration of LVAD support. Presence of AR led to a significantly higher expression of IFN-γ in LVAD patients [96].

Changes in aortic blood flow dynamics following LVAD support also induced changes in the aortic wall, which contributed to the development and progression of AR. Westaby et al. examined the aorta of seven individuals undergoing non-pulsatile axial-flow LVAD support. After 90 days of support, aortic wall atrophy was evidenced by a decrease in medial aortic thickness, medial smooth muscle cell number, and elastin content [97].

Although aortic root dilatation may not be large in magnitude, small amounts of dilatation with concomitant changes in wall elasticity and chronically high diastolic aortic pressures may promote valve malcoaptation and AR development. 

Ambardekar et al. compared aortic tissue collected at CF-LVAD implant and subsequently at HT from 22 patients and showed aortic remodeling and fibrosis after CF-LVAD that correlated with the duration of support [98].

Aortic wall morphometry, fibrillar collagen content, whole-transcriptome profiling by RNA sequencing, and immunohistochemistry were performed to evaluate CF-LVAD-mediated changes in aortic mRNA and protein expression. 

The authors found a significant increase in the thickness of the collagen-rich adventitial layer from pre- to post-LVAD implantation. There was also an increase in intimal and medial mean fibrillar collagen intensity from pre-LVAD to post-LVAD. The magnitude of this increase in fibrosis was greater among patients with longer durations of CF-LVAD support. CF-LVAD led to profound downregulation in the expression of extracellular matrix-degrading enzymes, such as MMP-19 and ADAMTS4, whereas no evidence of fibroblast activation was noted, suggesting that fibrosis is mainly due to suppression of extracellular matrix-degrading enzyme expression [98].

Similarly, significant regulation of mRNAs involved in extra-cellular matrix and collagen fiber organization in response to the implantation of LVAD was observed by Doulha et al. [99]. Paired aortic samples obtained at the time of LVAD implantation and at the time of HT were examined for mRNA/miRNA profiling. The number of differentially expressed genes shared between samples before and after LVAD support was 277. The whole-miRNome profile revealed 69 differentially expressed miRNAs. Gene ontology (GO) analysis identified that LVAD predominantly influenced genes involved in extracellular matrix and collagen fibril organization. Integrated mRNA/miRNA analysis revealed that potential targets of miRNAs dysregulated in explanted samples are mainly involved in GO biological process terms related to dendritic spine organization, neuron projection organization, and cell junction assembly and organization. The authors also found differentially expressed genes participating in vascular tissue engineering as a consequence of LVAD duration. Changes in aortic miRNA levels demonstrated an effect on molecular processes involved in angiogenesis [99].

## 6. Reverse Remodeling after LVAD Implantation

Levin et al. firstly demonstrated that mechanical unloading with LVAD might induce normalization of the end-diastolic pressure–volume relationship even in end-stage failing hearts, thus introducing the concept of reverse remodeling [100]. Among all therapeutic options for HF, LVAD support is associated with a higher degree of reverse ventricular remodeling not only through mechanical unloading, but also through improvement in cardiac output and blood pressure, as well as an attenuation of neurohormonal alterations [101]. LVAD support has been associated with significant improvement in left ventricular shape, with a reversal of left ventricular dilation and normalization of ventricular geometry [102]. The degree of reverse remodeling can be significant enough in some patients that LVAD explantation may be considered [103,104].

Reverse remodeling is associated with substantial cellular and molecular changes in the failing myocardium. Cardiomyocyte hypertrophy is a compensatory response to hemodynamic overload and neurohormonal activation and is characterized by a switch to a fetal gene expression profile and may become maladaptive. Mechanical unloading with LVADs reduces cardiomyocyte size and the myocardial expression of fetal gene isoforms [105]. Many signaling pathways, including GATA binding protein 4 (GATA-4), mitogen-activated protein kinases (MAPKs), extracellular signal-regulated kinase (ERK)-1/2 and c-Jun N-terminal kinase (JNK)-1/2, have been shown to be downregulated during LVAD support [106,107]. 

Previous studies on cardiomyocytes isolated from explanted hearts before and after LVAD support showed significant improvement in contractile properties, mainly due to an increase in calcium cycling and sarcoplasmatic reticulum calcium content [108], and normalization of calcium cycling genes including Na/Ca exchanger (NCX), sarcoplasmic endoreticular Ca^2+^ ATPase (SERCA), ryanodine receptor (RyR) 2, and protein kinase A-mediated hyperphosphorylation of RyR2 [109,110].

LVAD unloading also induces upregulation of β-adrenergic receptor density and increases the responsiveness to β-adrenergic stimulation [43]. 

LVAD supports have beneficial effects on myocardial metabolism as well. Even if LVAD support reduces mitochondrial number, it improves mitochondrial structure and function [111] and reduces mitochondrial oxidative stress [112]. HF is characterized by a fetal pattern of substrate with enhanced glycolysis and a reduction in fatty acid oxidation. LVAD support improves energetics through favorable changes in metabolic gene expression that improve fatty acid oxidation [113].

## 7. Right Ventricular Failure after LVAD Implantation

Right ventricular failure (RVF) is one of the main complications after LVAD implantation, occurring in at least 10% of patients [114], and it is associated with a poor outcome [115].

RVF post-LVAD implantation is due to multiple factors including increased LV cardiac output leading to increased venous return (preload) to the RV, left shift of the interventricular septum leading to decreased contribution to RV contraction, tachyarrhythmias, tricuspid valve regurgitation, and patient-related risk factors [114].

The beneficial effects of LVAD support that induce reverse remodeling of the LV only partially extend to the RV [116]. LVAD support can reduce RV afterload, but it does not reduce RV preload, and RV preload is frequently increased due to excess LVAD speed and flow and a patient’s hypervolemia. On the other hand, normalization of circulating neurohormones and restoration of myocardial beta-adrenergic responsiveness can be beneficial for both the LV and the RV. 

LVAD support entails positive hemodynamic changes, such as restoration of cardiac output and reductions in left ventricular and pulmonary arterial pressures. The decreased pulmonary arterial pressure due to a reduction in left-sided filling pressures and long-term remodeling of fixed pulmonary hypertension reduces pulmonary vascular resistance and improves RV afterload and function [117].

The LV unloading also improves RV function by decreasing functional mitral valve regurgitation and reversing an excessive shift of the septum into the RV [118].

On the other hand, changes in preload and alterations in ventricular interdependence through pericardiotomy and interventricular septal function play a central role in RVF pathophysiology. LVAD support decreases RV afterload, but it also raises RV preload as a consequence of increased LV output. The RV may increase the stroke volume to match the new left-sided supported cardiac output via a Frank–Starling mechanism, but the capacity of the RV to handle a higher preload depends on the pre-existing RV functional reserve. The higher preload can induce RV distension and tricuspid annular dilatation, thus worsening preexisting tricuspid regurgitation. Tricuspid regurgitation can also be exacerbated by tricuspid valve tethering due to leftward septal shift upon LV decompression with the LVAD support, further aggravating RV pressure/volume overload [119]. 

In summary, if LVAD delivers more volume than the RV can accommodate, the result is RV pressure/volume overload, eventually resulting in RVF.

To date, there are not specific biomarkers for the diagnosis and prediction of RVF. Henning et al. suggested a possible correlation between elevated pre-operative inflammatory and neurohormonal markers such as procalcitonin and BNP levels with the onset of right ventricular failure post-LVAD implantation [120].

Kato et al. showed that ECM markers decreased in patients with LVAD without RVF, while serum levels of MMP-2, TIMP-1, TIMP-4, and OPN remained elevated in patients with RVF [121]. Multivariate analysis identified that the right ventricular stroke work index (RVSWI) circulating BNP and OPN were significantly associated with RVF. Additionally, OPN that is inversely correlated with RVSWI and OPN levels >260 ng/mL distinguish patients who develop RVF from those without RVF [121].

Williams et al. analyzed the mRNA of RV samples from three groups of patients: (i) patients with LVAD and normal RV function; (ii) patients with LVAD and mild RVF; (iii) patients with LVAD and severe RVF, to determine the differential gene expression of key proteins [122]. They did not observe differences in protein expression between the groups. However, they reported a non-significant two-fold increase in SPARC-related modular calcium binding 2 protein in patients supported with LVAD and mild and severe RVF.


**LVAD**

**Biomarkers**
InflammationTNF-α (14, 15, 17, 18, 19, 23, 25), IL-6 (16, 19, 27, 29, 30), IL-1β (19, 35), Fas (19), FLICE (19), BNP (20, 24), IL-8 (21, 30), MCP-1 (21), interferon γ-induced, protein (21), CRP (21, 24, 30, 39), CD95 (28), NLR (31, 32), KLF-4 (33), TGM2 (35), MRC1 (33)FibrosisCollagen (43,45), MMP-2 (44), MMP-9 (44), MMP1 (45), TIMP-1 (45), ST2 (46–52), Galectin-3 (54–68)Bleeding eventsvWF (69–79) Aortic valve regurgitationα-SMA (96), CD68 (93), TGF-β (95), MMP-9 (96), actin (96), OPN (96), IFN-γ (96), IL-1 beta (96), TNF-α (96), MMP-2 (96), TGF-β (96), MMP-19 (98), ADAMTS4 (98)Reverse remodelingGATA-4 (105, 106), MAPKs (105, 106), (ERK)-1/2 (105, 106), JNK-1/2 (105, 106)Right ventricular failureProcalcitonin (119), BNP (119), MMP-2 (120), TIMP-1 (120), TIMP-4 (120), OPN (120)

## 8. Cardiovascular Mortality

Cellular and molecular pathways activated by LVAD implantation may have a significant impact on cardiovascular mortality. The elevation of inflammation markers early after LVAD implantation is correlated with higher mortality. [30]. Caruso et al. showed that LVAD induces systemic inflammation and the production of several cytokines such as IL-6, IL-8, and C-reactive protein (CRP), which are related to the development of multi-organ failure in the postoperative period [30]. Pre-operative elevated procalcitonin levels have been associated with RVF after LVAD implantation [120,123]. 

Previous studies demonstrated that natriuretic peptide circulating levels and mRNA expression in myocardial tissue decrease after LVAD implantation and reach a steady state at approximately 1 to 3 months post-implantation. BNP was measured at baseline at during the follow-up in plasma of a cohort of 103 patients undergoing CF-LVAD implantation. Following LVAD implantation, BNP levels significantly reduced; however, they remained abnormal (>35 pg/mL) and above 100 pg/mL in most patients [124]. Cox regression analysis revealed that higher baseline and follow-up BNP levels were not associated with increased risk of death at 180 days. In the univariate analysis, 90-day BNP, but not baseline BNP, was significantly associated with the combined death/hospitalization outcome 180 days after LVAD implantation; however, this significance was not preserved after adjusting for multiple covariates [124]. Likewise, another study reported on serial BNP measurements in 83 patients before and at 30, 60, and 90 days after LVAD implantation [125]. The authors found that the BNP levels at 60 days were associated with worse survival using univariate analysis; however, BNP failed to be a predictive risk factor for mortality using multivariate analysis [125]. Another study conducted on 72 continuous-flow LVADs revealed that NT-proBNP levels peaked 3 days after surgery and subsequently decreased, still remaining above the upper limit of normal at discharge in all patients. Additionally, patients with complicated postoperative courses had higher early postoperative elevations [126].

It remains controversial whether the development of AR after LVAD implantation has an impact on clinical outcomes and survival. Some reports showed a decreased survival and increased adverse cardiac events, while others failed to demonstrate any direct association. Previous studies showed worsening of HF symptoms, greater rates of AV repair, and the need for heart transplantation for refractory HF due to severe AR [82,127,128].

On the contrary, Cowger et al. found no significant impact of AR development on survival in 166 implanted patients, and a similar rate of mitral regurgitation, RV dysfunction, pump thrombosis, and device exchange between patients who developed AR and those who did not [129]. Similarly, Holley et al. showed a 15.2% prevalence of at least moderate AR in 237 patients followed for 5 years with no impact on survival [130]. 

A recently published report from the Interagency Registry for Mechanically Assisted Circulatory Support (INTERMACS) showed that patients with moderate/severe AR had significantly lower survival at 5 years compared to those who had no mild AR, and differences in survival persisted after adjustment for age, INTERMACS profile, and chronic kidney disease. Patients who developed significant AR in the first year after LVAD implantation had lower freedom of hospitalization at 2 years, without significant differences in stroke, arrhythmia, and bleeding events [131]. An analysis of the STS INTERMACS registry showed that patients with persistent RVF at 6-month follow-up had inferior 1- and 3-year survival compared with patients with no RVF. [132]. Late RVF is associated with poor long-term survival compared with patients with early RVF or no RVF, and mortality is 80% at 2 years from the diagnosis of late RVF [133]. Additionally, patients with late RVF bridged to HT have a significantly reduced 5-year survival and an increased rate of complications such as primary graft dysfunction, renal failure requiring hemodialysis, and infectious complications compared to patients without RVF [134]. RVF is also associated with an increased risk of adverse events such as gastrointestinal bleeding and stroke [135,136]. Gastrointestinal bleeding is due to increased venous pressures causing intestinal mucosal congestion, hepatic congestion with associated coagulopathy, and increased risk of arteriovenous malformations due to intestinal hypoxia [137]. RVF also causes coronary sinus congestion that affects coronary perfusion, resulting in the development of RV ischemia and ventricular arrhythmias. Elevated venous pressures and decreased cardiac output to the liver, kidney, and gut can lead to increased volume expansion, ischemia, and inflammation, resulting in end-organ dysfunction and increased mortality [138].

## 9. Role of Nutrients in LVAD

LVAD patients experience several post-operative issues, including malnutrition [139,140]. Some patients develop an impaired nutritional state after LVAD implantation upon experiencing gastrointestinal disturbances that limit food intake (early satiety, decreased appetite, intermittent nausea, and vomiting) [141], likely due to the luminal compression exerted by the device [142], or bleeding [143]. Some others, instead, experience an exacerbation of a pre-existing undernourished or malnourished state after LVAD surgery. In CHF patients, in fact, blood stasis in systemic and pulmonary circulation causes intestinal hypoperfusion or interstitial edema that results in impaired gastrointestinal absorption [144,145].

**Figure 2:**
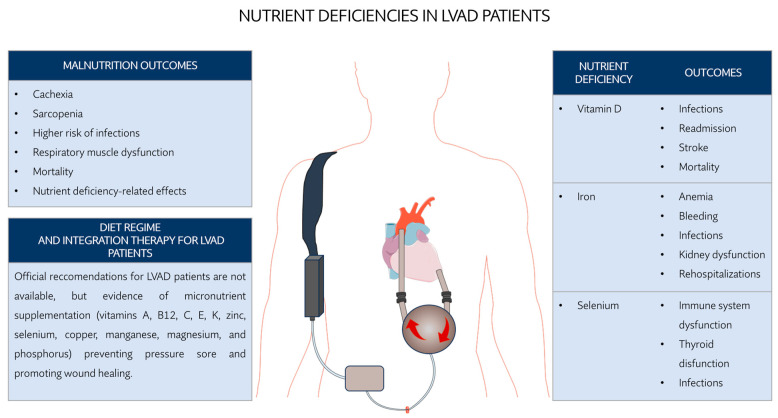
Effect of nutrient deficiencies in LVAD.

If not corrected, malnutrition eventually leads to cachexia and sarcopenia [141], as well as nutrient deficiency and the related outcomes [146,147,148]. There is evidence that an impaired nutritional status is associated with a higher risk of developing infections after LVAD implantation [149,150], respiratory muscle dysfunction, and more severe outcomes, including death [149,151], especially if malnutrition is preliminary to the device implantation [147] (Figure 2, Table 1). Micronutrient supplementation (vitamins A, B12, C, E, K, zinc, selenium, copper, manganese, magnesium, and phosphorus) before and after LVAD implantation has been recognized to be effective in preventing pressure sores and in promoting wound healing [152]. 

While the importance of maintaining an adequate nutritional state is increasingly recognized in clinical practice, the current nutritional guidelines do not provide any specific recommendations for the management of LVAD patients’ nutrition [145] and generally follow recommendations for healthy individuals [139]. Furthermore, there is controversial evidence about the effectiveness and safety of the administration of some nutrient supplements to treat deficiencies that will be discussed in the next paragraphs. 

Iron deficiency is the most common cause of anemia diagnoses in patients subjected to LVAD implantation [147]. Patients exhibiting anemia and iron deficiency have low life expectancy and enhanced frequency of disturbances such as frequent bleeding, infections, kidney dysfunction, and rehospitalizations [153]. The administration of oral iron is widely used for treating such deficiency [146]. However, in some cases, this treatment has been reported to be irrelevant in terms of clinical impact or even harmful because of a higher risk of GI bleeding [146]. In these cases, intravenous IV iron infusions have been suggested as a more suitable and safer option [146], except for ill or septic LVAD patients where such formulation might increase the risk of infection [139]. 

Vitamin D deficiency is frequently encountered in patients with congestive HF and has been associated with a higher risk for postoperative driveline infections, stroke, and mortality in CHF patients undergoing LVAD surgery [154,155,156,157]. Optimal levels for vitamin D supplementation range from 400 to 2000 IU daily, with 4000 IU per day being the upper daily limit indicated by several health organizations including the Institute of Medicine (USA) and the European Food and Safety Authority [158]. However, the preoperative supplementation for LVAD patients was reported to not be effective because small to no significant differences were obtained upon either daily, weekly, or high-dose monthly supplementation of vitamin D, as reported in the panel of studies listed in Busa et al., 2020 [156]. Increasing levels of circulating 1,25-dihydroxyvitamin D (1,25(OH)2D), the active form of vitamin D, have been reported as a secondary consequence of the suppression of FGF-23 levels and the improvement in kidney function following LVAD implantation [159]. 

Other deficiencies associated with heart diseases (e.g., acute coronary artery disease) is selenium, an essential nutrient used for the enzymatic activity of selenoproteins [160]. In LVAD patients, who are frequently immunosuppressed and exhibit thyroid dysfunction [158], these proteins are likely to play a key role by enhancing antioxidant defense, thyroid functioning, and immunity responses [160]. Differently from other deficiencies, selenium supplementation in LVAD patients has been reported to be effective in compensating for low selenium levels and promoting slightly better physical functioning, but no detectable improved quality of life [148]. However, further investigation is needed given the limited evidence of its impact on clinical outcomes.

Overall, overcoming nutrient deficiency in LVAD patients is expected to enhance patient care. While LVAD implantation per se has revealed an improvement in the nutritional state due to the putative resolution of HF-related dysfunctions [161], a better understanding of how diet and nutrition might impact and prevent severe outcomes in HF and LVAD therapy is emerging. Specifically, some nutrients such as amino acids (i.e., glutamine; arginine), antioxidant vitamins (i.e., vitamin E), omega-3 fatty acids, and minerals (i.e., zinc; copper; selenium) have been demonstrated to modulate the immune system and body’s response to inflammation in different populations of non-LVAD immunosuppressed patients, thus defining the so-called immunonutrition [162]. The European Society for Clinical Nutrition and Metabolism (ESPEN) has defined guidelines [163] for home enteral nutrition with specific consensus recommendations or exclusion criteria for immunonutrient supplementation in surgical, injured, or critically ill patients. Despite the amount of evidence supporting the efficacy of supplementation of each of these nutrients, it is still not clear why, in some groups of patients, the same therapy is clinically ineffective or even harmful [162,163]. New research and clinical trials are therefore needed in view of personalized medicine. Given the absence of specific nutritional evaluation and management of LVAD surgical patients, clinical trials such as “Enhanced Nutritional Optimization in LVAD Trial (ENOL)” (Identifier: NCT05655910) have recently started with the aim of evaluating the beneficial effects of the use of a preoperative immune-modulating diet (i.e., Ensure Surgery Immunonutrition shake) in patients undergoing LVAD implantation. The first outcomes of the study are expected by September 2024.

## 10. Conclusions

LVAD support markedly improves survival and quality of life in most patients with end-stage HF, as indicated by the ELEVATE Registry [164]. However, the risk of mortality remains high even after implantation, despite continuous improvements in device technology and surgical experience. Some patients undergoing LVAD implantation survive but continue to have poor quality of life due to refractory or recurrent HF symptoms, failure to recover from major cardiac surgery, or device-related complications such as bleeding, infections, stroke, and pump malfunction requiring frequent re-admissions that markedly reduce the cost-effectiveness of this procedure [165]. Further studies are required to ascertain the cellular, molecular, and genetic pathways underlying both positive and negative effects elicited by LVAD support. 

## Figures and Tables

**Table 1:** Effect of nutrient deficiency and related supplementation in LVAD.

Nutrient Deficiency	Symptoms Resulting from Nutrient Deficiency	Treatment/Tested Dosage	Efficacy of Supplementation	Secondary Outcomes
Iron	Anemia; bleeding, infections, kidney dysfunction, and rehospitalizations [153]	Oral iron		Increasing risk of GI bleeding
		Intravenous iron	Restoring iron serum levels	Increased risk of infection in septic patients
Vitamin D	Higher postoperative infection risk ad rate of readmission [154]; Stroke and mortality [155]		Not effective	
Selenium/ Minerals [148]	Immune system dysfunction; increased susceptibility to infections	300 mcg of selenium orally the evening before surgery, followed by a high dose of intravenous selenium supplementation (3000 mcg after anesthesia induction, 1000 mcg upon intensive care unit [ICU] admission, and 1000 mcg daily in the ICU for a maximum of 14 days	Restoring serum selenium concentrations	Acute renal failure in 30% of patients
			Slightly better physical functioning	Same frequency of nosocomial infections and mortality
				Longer ICU and hospital LOS
			data	data
